# Structural bases of peptidoglycan recognition by lysostaphin SH3b domain

**DOI:** 10.1038/s41598-019-42435-z

**Published:** 2019-04-12

**Authors:** Paweł Mitkowski, Elżbieta Jagielska, Elżbieta Nowak, Janusz M. Bujnicki, Filip Stefaniak, Dorota Niedziałek, Matthias Bochtler, Izabela Sabała

**Affiliations:** 1 International Institute of Molecular and Cell Biology in Warsaw, Warsaw, Poland; 2 Laboratory of Bioinformatics, Institute of Molecular Biology and Biotechnology, Faculty of Biology, Adam Mickiewicz University, Poznan, Poland; 3 Institute of Biochemistry and Biophysics Polish Academy of Sciences, Warsaw, Poland

## Abstract

*Staphylococcus simulans* lysostaphin cleaves pentaglycine cross-bridges between stem peptides in the peptidoglycan of susceptible staphylococci, including *S. aureus*. This enzyme consists of an N-terminal catalytic domain and a cell wall binding domain (SH3b), which anchors the protein to peptidoglycan. Although structures of SH3bs from lysostaphin are available, the binding modes of peptidoglycan to these domains are still unclear. We have solved the crystal structure of the lysostaphin SH3b domain in complex with a pentaglycine peptide representing the peptidoglycan cross-bridge. The structure identifies a groove between β1 and β2 strands as the pentaglycine binding site. The structure suggests that pentaglycine specificity of the SH3b arises partially directly by steric exclusion of Cβ atoms in the ligand and partially indirectly due to the selection of main chain conformations that are easily accessible for glycine, but not other amino acid residues. We have revealed further interactions of SH3b with the stem peptides with the support of bioinformatics tools. Based on the structural data we have attempted engineering of the domain specificity and have investigated the relevance of the introduced substitutions on the domain binding and specificity, also in the contexts of the mature lysostaphin and of its bacteriolytic activity.

## Introduction

Most peptidoglycan hydrolases, particularly those of phage origin, have modular structure and are built of at least one catalytic and one cell wall binding domain (CBD)^1^. So far, several different CBDs have been described in the literature, such as LysM^2^, Cpl-1^3^, Cpl-7 domain^4^ and the family of bacterial SH3b domains^1,5^. Different CBDs recognize and bind noncovalently to various elements of the cell walls, like the peptidoglycan sugar backbone (LysM)^6^, choline (Cpl-1)^3^, teichoic acids (R domain)^7^, and peptidoglycan cross-bridges (SH3b)^8^. The specificities of CBDs vary considerably. Some are specific only for certain serovars, as in *Listeria* phage endolysins^9,10^, others recognize peptidoglycan elements characteristic for a whole genus, as SH3b domains in a variety of different peptidoglycan hydrolases.

SH3b domains that are present in many staphylococcal phage endolysins and some autolysins, are well conserved^11,12^. These domains are necessary for accurate cell wall recognition^8,13,14^ and have been shown to recognize and bind glycine cross-bridges characteristic for most staphylococci^15^. By binding to receptors in the bacterial cell walls SH3b domains might regulate the activity of peptidoglycan hydrolases^16^. In our previous work we have demonstrated that addition of lysostaphin SH3b domain to the catalytic domain of LytM, an autolysin from *S. aureus*, made the enzyme more salt-tolerant^17^.

Structures of SH3b domains have so far been determined for Ale-1^8^ and lysostaphin^18^, both in the absence of their ligands. Although regions of SH3b domains that contribute to the substrate specificity have been defined by mutagenesis experiments, the precise interactions between SH3b domains and peptidoglycan are still unclear. A better understanding of these interactions is desirable, because it may allow for engineering of the domain to broaden the range of targeted bacteria but also to overcome lysostaphin resistance, which is caused by introduction of serines into the cross-bridge^19,20^.

Here we present the first structure of bacterial SH3b domain in complex with pentaglycine and characterize binding of the domain to other elements of peptidoglycan using biophysical and biochemical methods supported by bioinformatics tools and molecular dynamics. We show that these interactions are crucial for the function of lysostaphin and we attempt structure based engineering of the domain specificity.

## Results

### Overall structure of the lysostaphin SH3b domain

**Figure 1:**
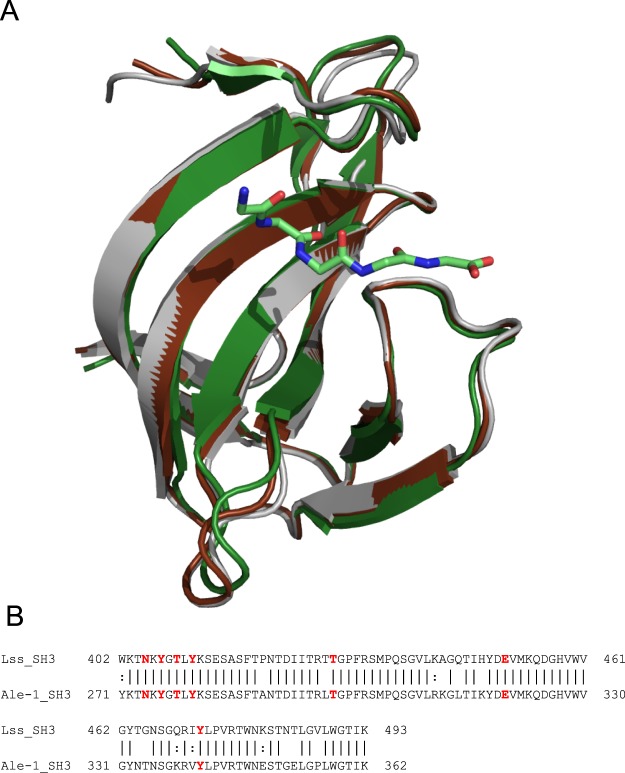
(**A**) Structural comparison of the lysostaphin SH3b domain in complex with the pentaglycine peptide (dark green; PDB 5LEO) with the SH3b domains of Ale-1 (gray; PDB 1R77) and the apo-lysostaphin (brown; PDB 4LXC). (**B)** Alignment of amino acid sequence of SH3b domains from lysostaphin and Ale-1. Residues involved in interactions with pentaglycine are marked as red.

The structure of the SH3b domain of lysostaphin is very similar to its counterpart in the previously determined Ale-1^8^ structure, as could be expected based on the high degree of sequence similarity between these domains (84% sequence identity over 90 residues) (Fig. 1).

**Figure 2:**
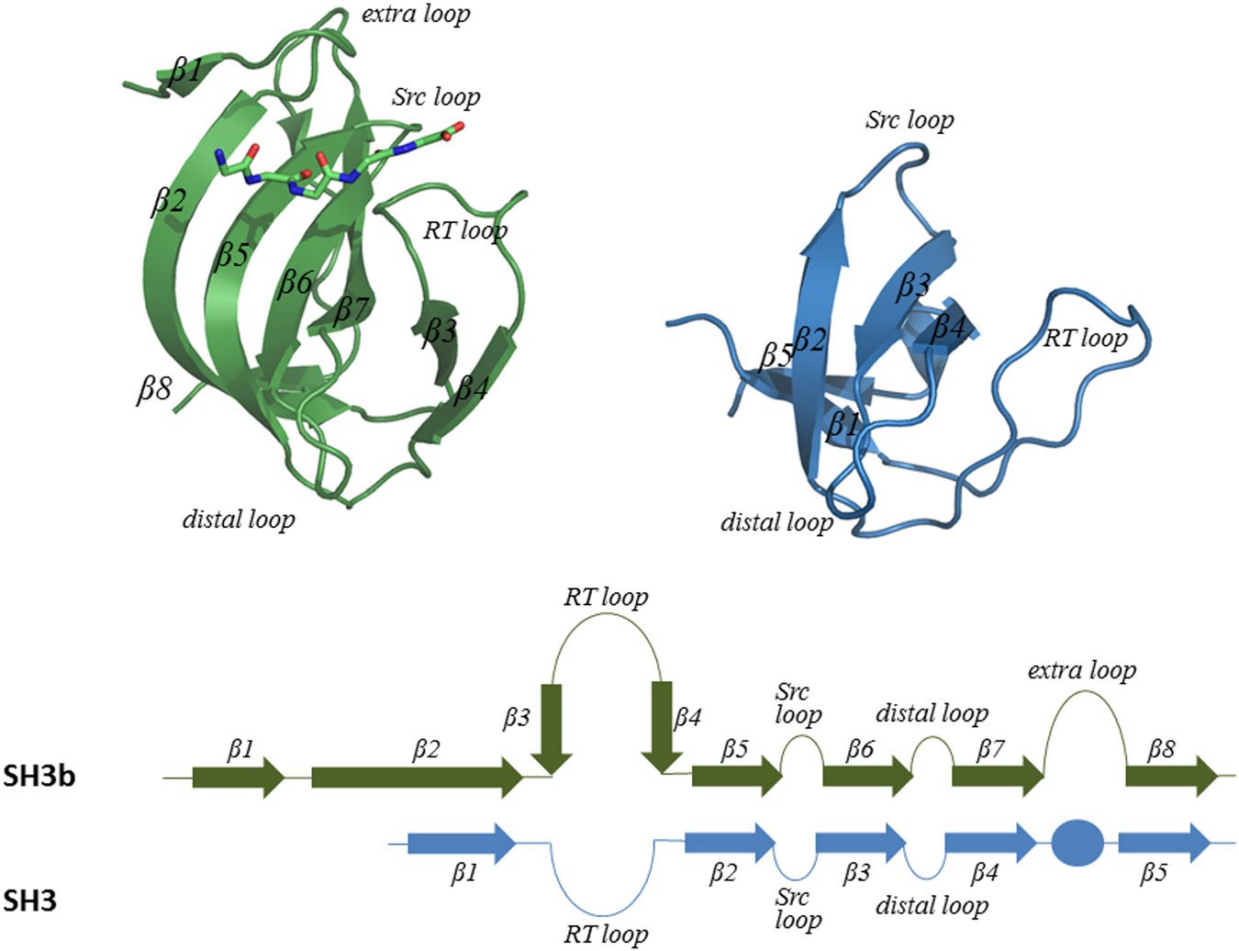
Cartoon representation and topology of the lysostaphin SH3b (green) and eukaryotic SH3 domains (blue).

As reported already for Ale-1, the prokaryotic SH3b domains are built of eight β-strands (β1–β8) that can roughly be divided into two sheets packed at the right angle against each other. The first sheet is made from β5–β7 and the N-terminus of β2, whereas the second sheet is made from β3-β4, β8 and the C-terminus of β2. The strands β1, β3 and β4 are unique to Ale-1 and lysostaphin SH3b domains, whereas the other β-strands are present in both, eukaryotic and bacterial domains (Fig. 2). The strands β3 and β4 are located in the so-called RT loop (following the nomenclature of the Ale-1 structure description). In canonical SH3 domains, this loop has been implicated in domain specificity^21,22^. Mutational data also support the role for the SH3b domain of Ale-1 in sequence specificity^8,23^. They implicate strands β1 and β2 in the binding, since a fragment lacking these strands is drastically impaired in specific binding to lysostaphin susceptible peptidoglycan, but not in background binding to other types of peptidoglycan^8^.

**Figure 3:**
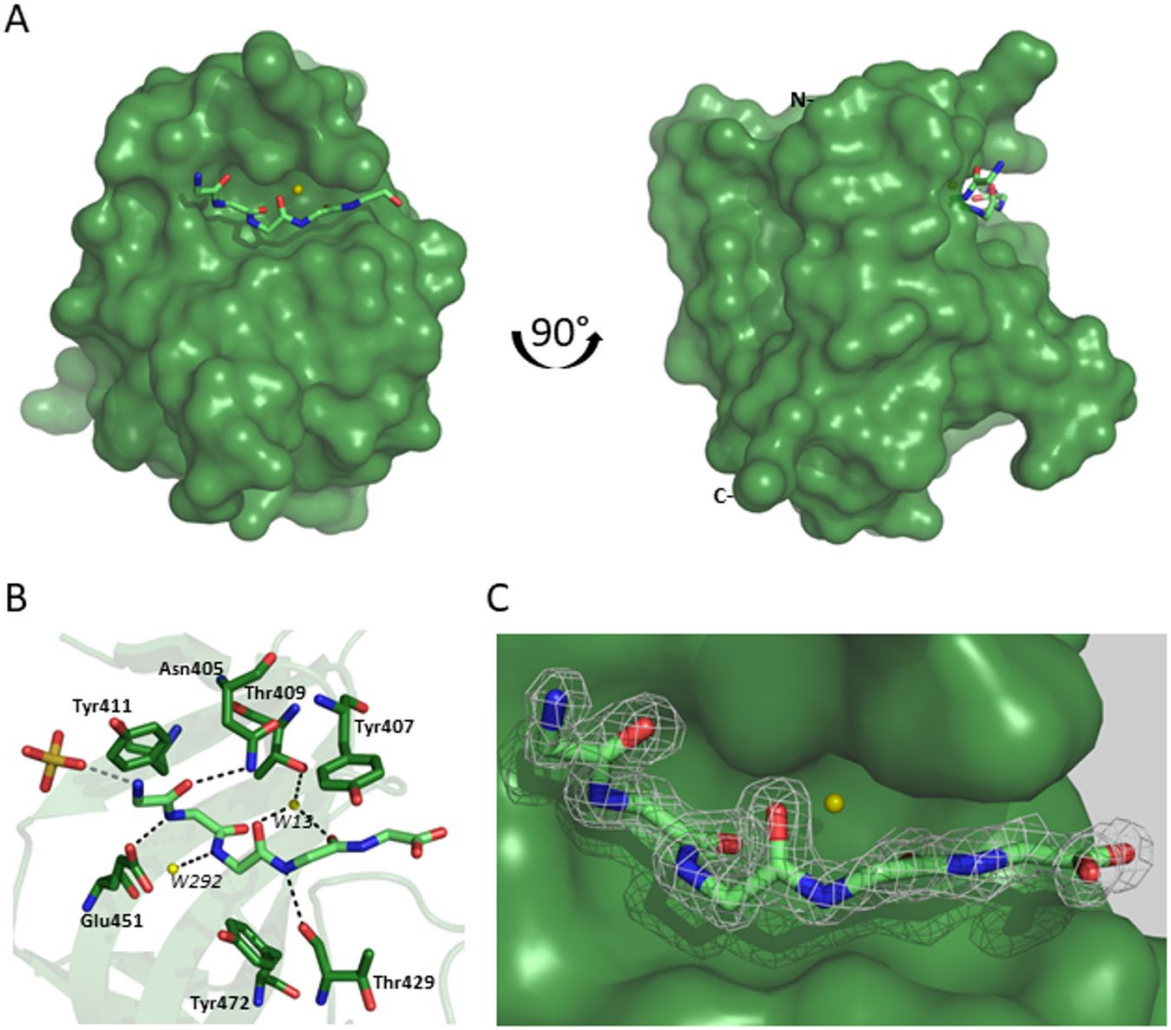
Structure of lysostaphin SH3b domain in complex with pentaglycine. (**A**) front view, (**B**) Close up view of residues interacting with the pentaglycine peptide, (**C**) Calculated composite omit electron density map of pentaglycine at 1 σ.

The overall structure of the lysostaphin SH3b domain in the presence of the pentaglycine ligand is very similar to the structure of the domain in the context of the full length protein, but in the absence of ligand^18^ (r.m.s.d. value 0.7Å, main chain). The new lysostaphin SH3b structure is also very similar to the structure of *S. capitis* Ale-1 (r.m.s.d. value 0.9Å, main chain). The only variations are found in loop regions, mainly distal and extra loops. Also no differences were observed between the two molecules present in the asymmetric unit, the r.m.s.d. of 0.61Å was calculated for all atoms of chains A and B, and 0.22Å between two ligands (chains G and H). These comparisons indicate that there are no significant differences between the structures of Ale-1 and lysostaphin SH3b domains, and that pentaglycine binding does not induce significant structural change (Fig. 1).

### Pentaglycine binding mode

The structure of the SH3 domain of lysostaphin (PDB ID: 5LEO) is informative beyond what could have been predicted by bioinformatic analysis alone, because it elucidates the binding mode of pentaglycine to the SH3b domain at high resolution, and with a good definition of the bound peptide along its entire length (real space correlation coefficients >0.85 for all five glycines). The side and bottom walls of the binding grove are built of side chains of residues Asn405, Tyr407, Thr409, Tyr411, Thr429, Gly430, Pro431, Phe432, Met435, Glu451, Met453, and Tyr472. The bound pentaglycine molecule adopts an extended conformation, in between strands β1 and β2 on the one side and the RT loop on the other side. As both elements of the SH3b domain have previously been implicated in peptidoglycan binding in the case of the Ale-1 SH3b domain^8^, it is likely that the pentaglycine binding mode that is observed in the crystals is physiologically relevant. The pentaglycine peptide runs approximately parallel to β1 and anti-parallel to β2, but there are no β-strand specific main chain hydrogen bonding contacts, because the distance is too large. Instead of interacting with the main chain, the pentapeptide interacts with side chains from both β1 and β2 and from the RT. Due to the lack of backbone interactions, pentaglycine can adopt a non-canonical secondary structure characterized by carbonyl groups that are neither parallel (as in α-helices) nor alternatingly antiparallel (as in β-strands).

### Structural determinants of interactions with pentaglycine

In the crystal structure, Gly1 exposes an N-terminal α-amino group, which forms a salt bridge with a sulfate or phosphate ion from the crystallization buffer, which is also involved in inter-subunit contacts (that are different for the two SH3b monomers in the asymmetric unit). In peptidoglycans the N-terminal amino group of Gly1 should be connected in amide linkage with the carboxylate group of the D-Ala residue of the stem peptide, therefore this interaction is a crystal artifact. However, as the sulfate or phosphate is bound on the surface, its presence shows that there is space on the SH3b domain for the stem peptide that would be upstream of the pentaglycine in a peptidoglycan context. The carbonyl oxygen atom of Gly1 accepts a hydrogen bond (donor-acceptor distance 3.1Å) from the side chain carboxamide of Asn405, located at the end of β1. The Asn405 carboxamide is similarly close to the carbonyl oxygen atoms of Gly2 and Gly3, but the geometry is not compatible with hydrogen bonding. The carbonyl oxygen atom of Gly2 accepts instead an indirect, water mediated hydrogen bond from Thr409. Gly2 is additionally held by a hydrogen bond from its NH group to the carboxylate group of Glu451, which is anchored in a region of the SH3b domain not previously implicated in peptidoglycan binding. Moreover, the amide bond between Gly1 and Gly2 packs at van-der-Waals distance against the side chain of Tyr411 of β2. The conformation of Gly3 appears to be primarily determined by its covalent linkage to the upstream and downstream residues of the pentapeptide. The Gly3 NH group points towards solvent and hydrogen bonds to a water molecule. The carbonyl group comes within hydrogen bonding distance to the ND2 atom of Asn405, but the geometry does not appear to be compatible with hydrogen bond formation. The position and orientation of the carbonyl group of Gly3 is rather determined by the hydrogen bond that Gly4 NH donates to the main chain carbonyl oxygen atom of Thr429 of the RT loop. The carboxyl oxygen atom of Gly4 accepts a water-mediated hydrogen bond to Thr409. The threonine residue and water molecule that are involved in anchoring the carbonyl oxygen of Gly4 are the same ones that also anchor the carbonyl oxygen atom of Gly2. The threonine residue and water molecule therefore appear to have a central role in defining the pentapeptide conformation. Gly5 stacks against the aromatic ring of Tyr407, but it does not engage in hydrogen bonding interactions with the SH3b domain that is bound to, but forms a salt bridge between its carboxylate group and an arginine of a neighboring molecule in the crystal (Fig. 3).

### Characterisation of binding

#### MST (microscale thermophoresis)

Fluorescently labelled LssSH3b was incubated with GGGGG, GGSGG or AQKAA (stem peptide). Unspecific binding was monitored using peptide GRGDS. Specific interactions were observed for pentaglycine but due to limited solubility of this peptide and weak affinity the full binding curves necessary for calculation of K_d_ could not be recorded. However, it could still be concluded that interactions of LssSH3b domain with short peptidoglycan fragments are weak (milimolar range) for both the stem peptide and pentaglycine. Substitution of middle glycine with serine in GGSGG peptide reduced the interactions even further (Supp. Fig. S1).

#### Scatchart analysis

To evaluate the strength of SH3b domain binding to whole cells Scatchard analysis was performed. The Scatchard plots of different strains of *S. aureus* yielded straight lines (Supp. Fig. S2) from which a K_d_ was estimated to be in the micromolar range (Table 1). Substitution of one of the glycines in the bridge with serine (TF5311 strain) weakens the binding affinity as compared to parental stains with penatglycine bridge (TF5303 strain). For *S. aureus* mutants *femB* and *femAB* with shortened cross-bridges, the binding affinity could not be determined due to very weak signals.

**Table 1:** Results of Scatchard analysis of SH3b_GFP binding to various strains of *S. aureus*.

Strain (bridge structure)	K_d_(µM)	Number of SH3b molecules attached to single bacterial cell
8325-4 (GGGGG)	1,95 ± 0,28	0,65 ± 0,18*10^6^
TF5303 (GGGGG)	1,02 ± 0,63	0,46 ± 0,36*10^6^
TF5311 (GGSGG)	2,23 ± 0,03	0,36 ± 0,08*10^6^
*femB (GGG)*	*nd*	*nd*
*femAB (G)*	*nd*	*nd*

As defined numbers of *S. aureus* cells were used, we could calculate that at most between 10^5^ and 10^6^ SH3b_GFP could be bound to a single *S. aureus* cell (Table 1). These numbers correspond to the results of other estimation based on lysostaphin activity inhibition by preincubation of bacterial cells with Lss SH3b domain. The number of SH3b molecules bound to a single cell in those experiments ranged between 10^5^–10^6^, assuming the number of given cells and SH3b concentration which nearly completely reduced Lss activity (Supp. Fig. S3).

#### Bioinformatic analysis

The binding affinity of entire cells appeared to be much higher than the one of just isolated peptides representing cross-bridges, which suggests that SH3b domain might interact also with other than pentaglycine elements of the peptidoglycans. Considering the size of the domain and the high level of staphylococcal peptidoglycan crosslinking^24^, the SH3b domain may interact with the dense peptidoglycan network not only in the vicinity of the binding cleft for pentaglycine but in other areas of the protein surface as well.

**Figure 4:**
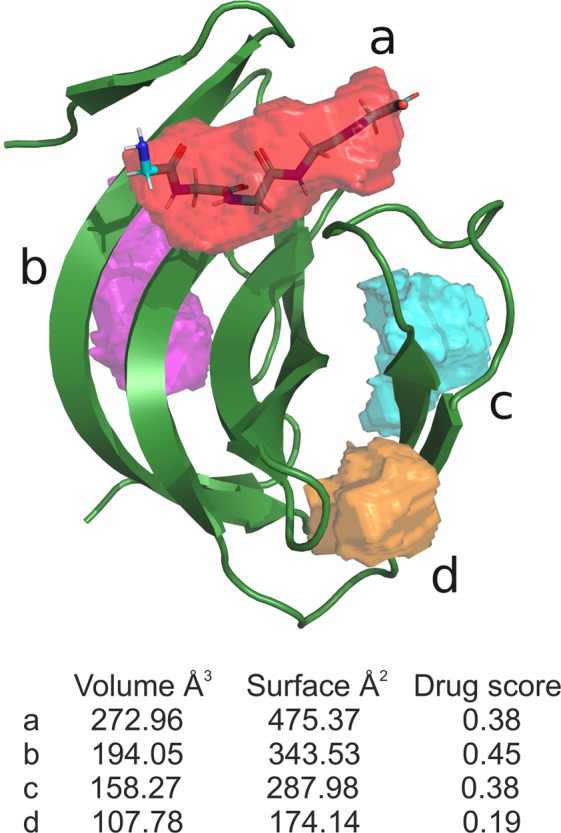
Peptide binding sites on the surface of lysostaphin SH3b domain predicted using DoGSiteScorer webserver.

To predict potential additional peptidoglycan interaction regions on the surface of the SH3b domain the DoGSiteScorer webserver was used^25^ (Fig. 4).

The main predicted binding pocket (pocket **a** in Fig. 4) with the highest volume, surface and drug score matches perfectly the binding groove occupied by pentaglycine in the complex structure. Three additional pockets (pockets **b**, **c**, **d** in Fig. 4) also scored highly suggesting that there is a very high probability of binding peptidoglycan network in those regions of the SH3b domain.

### Molecular modeling

To ensure the validity of the proposed binding mechanism and analyze further interactions of the SH3b domain with other elements of peptidoglycans, namely stem peptides, we have complemented our studies by combining molecular docking and Molecular Dynamics (MD) simulation techniques. During both types of modeling, we assumed a full flexibility of the ligand, having in mind that it may be reduced in case of the potentially more rigid PG network (for details, see “Molecular modeling of the SH3 domain-peptidoglycan complex” section in Materials and Methods).

Simulations with pentaglycine bound to the SH3b domain identically as in the 5LEO structure, revealed instability of this complex, due to a very high flexibility of the pentaglycine chain, what immediately lead to the dissociation of the pentaglycine molecule from the binding pocket already at room temperature. Similarly, stem peptides alone (not connected *via* pentaglycine bridge) were not able to remain bound with the SH3b protein domain at room temperature (data not shown).

**Figure 5:**
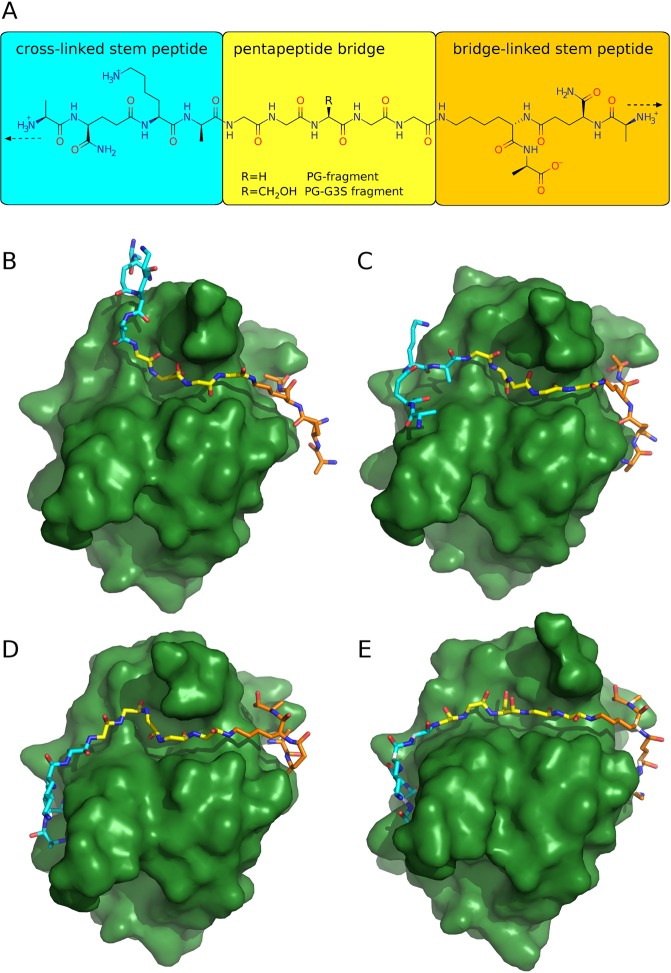
(**A**) Structure of ligand (PG fragment) used for modeling. Background colors (cyan for the cross-linked stem peptide, yellow for the pentapeptide bridge, orange for the bridge-linked stem peptide) corresponds to colors of the ligands’ main chain in the panels (B–E). Dashed arrows indicate directions of the peptidoglycan network. The terminal amino groups of the fragments used for modeling were charged, however in the muramoyl-linked stem peptide in the peptidoglycans, these groups are neutral (**B**–**E**). Predictions of the binding modes of PG-ligands with SH3b domain of lysostaphin: PG-fragment (**B**–**D**) and PG-G3S fragment (**E**). Poses B and E had been initially obtained using molecular docking and then confirmed as stable conformations by Molecular Dynamics simulations. Poses C and D have been obtained and confirmed as stable during Molecular Dynamics exploration of the conformational space of the PG-fragment upon binding SH3b.

Much stronger binding was predicted for the whole fragment of the peptidoglycan comprising pentaglycine linking two stem peptides. We docked a three-dimensional model of a PG fragment (Fig. 5A) to the crystal structure of the SH3b domain. To assess the stability of the predicted SH3b-ligand complex, MD simulations were performed. Simulations carried out at room temperature confirmed the stability of the complex predicted by molecular docking (Fig. 5B). In this pose, the pentaglycine bridge of the PG-fragment (Fig. 5, marked as yellow) remains within the binding pocket occupied by GGGGG in our crystal structure (PDB ID: 5LEO) and stem peptides are oriented toward predicted binding pockets.

To explore further the conformational space of the PG fragment upon interaction with the SH3b domain, we performed MD analyses at higher temperatures (Fig. 5C,D). Although the stem peptides of the PG fragment fluctuated significantly during the dynamical evolution of the SH3b-PG fragment complex already at room temperature (RT), the pentaglycine bridge remains within the binding pocket of the SH3b even at higher (*i.e*. >300 K) temperatures.

As can be seen in Fig. 5, the bridge-linked stem peptide always occupies the same part of the SH3b surface in a conformation maximizing the hydrogen bonding, which is located in a groove formed between β7 strand and RT loop. On the contrary, the cross-linked stem peptide can adopt different conformations interacting *via* hydrogen bonds with different parts of SH3b surface. The conformation of the cross-linked stem peptide mostly depends on the C1-Cα torsional angle on the Gly1 residue of the pentaglycine allowing this stem peptide to accommodate in the groove formed within the β1 strand (Fig. 5a) or in the located ~10Å away and biggest detected binding pocket (Figs 4b and 5b–d). The dynamical evolution between the PG-fragment conformers will be discussed in detail later.

Bioinformatic tools helped to analyze the consequences of substituting the middle glycine with serine, which is known mechanism of SH3b binding inhibition^20^. Replacing the Gly3 of the pentaglycine ligand by serine leads to: (i) a discernible steric hindrance by the hydroxymethyl side chain, (ii) induced stiffness in the middle of the ligand, and (iii) disturbance of the specific hydrogen bonds network between the SH3b and the bridge. Moreover, the Gly3 replacement by serine (iv) allows for intramolecular hydrogen bond interactions between the hydroxymethyl side chain and the main chain of the ligand. Such bonding increases stiffness and causes adverse confirmation of the GGSGG ligand. All these factors lead to a significant decrease in both specificity and stability of the SH3b-GGSGG complex with respect to SH3b-GGGGG, which is manifested by a hindered binding and immediate dissociation of the primary complex. Analogous effects were observed when the Gly3 residue was replaced by serine in the pentaglycine bridge of the PG fragment, though they did not lead to the dissociation of the complex. This additional stability was a result of the interactions (mostly *via* hydrogen bonds) between the SH3b domain and the stem peptides. These interactions also increase the affinity of the complex by playing a role of additional recognition sites. Moreover, the serine substitution in the middle of the pentaglycine bridge allows for intramolecular hydrogen bonding between the serine’s hydroxyl group and carbonyl groups of flanking glycines.

In summary, the Gly3 replacement by serine significantly influences the conformational properties of both bridge alone and PG-G3S fragment.

### SH3b mutagenesis

One of the concerns related to application of lysostaphin as an effective antimicrobial agent is resistance observed when cross-bridge glycines are replaced by other amino acids, in most cases serine at position 3. Such altered cross-bridges are much less prone to lysostaphin cleavage due to deceased susceptibility of such substrates to catalysis but also because of reduced binding of the SH3b domain^26^.

Our structure analysis reveals two main residues building the binding groove around Gly3 of the bound pentaglycine: asparagine 405 (Asn405) and tyrosine 472 (Tyr472). While Asn405 seems to play a major role in the pentaglycine binding, *in silico* substitution of Gly3 to serine indicates possible clashes also with the side chains of Tyr472. We assumed that shortening the side chain of Tyr472 might generate more space for accommodation of serine at position 3 in the cross-bridge, and replacing Asn405 with alanine could allow for binding peptide less flexible than pentaglycine. To test the relevance of Asn405 and Tyr472 in substrate recognition and binding these two residues were substituted with alanine in single and double variants. This mutagenesis was performed both on LssSH3b fused to GFP to characterize binding properties and on mature lysostaphin to test their effect on lytic activity of the enzyme. Activity and binding assays were performed on *S. aureus* reference strain but also mutants with serine introduced in the cross -bridge and cross-bridges shortened to three or one glycine.

### Effect of substitutions on SH3b binding

**Figure 6:**
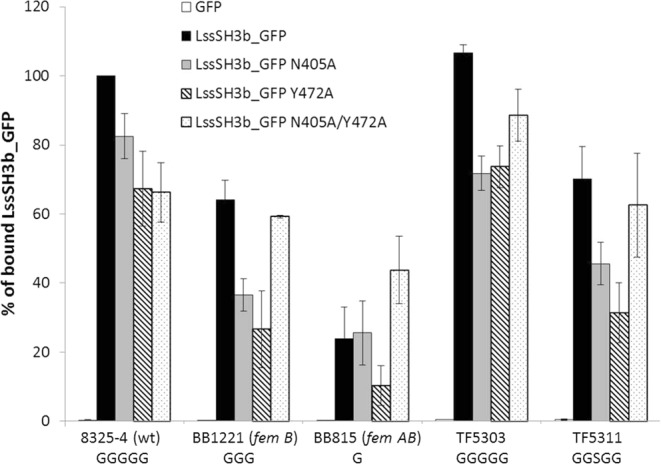
Fluorimetric binding assay of wild type LssSH3b_GFP and its variants (N405A, Y472A, N405/Y472) with various strains of *S. aureus* which represent wild type (pentaglycine) or altered cross-bridges. Results were normalized to the % of binding of wild type LssSH3b_GFP domain to wild type *S. aureus* NCTC 8325-4 (100%). The error bars represent standard deviation calculated for three experiments each run with triplicates.

Fluorimetric assays with LssSH3b_GFP fusion protein were run to characterize specificity of LssSH3b (lysostaphin SH3b domain) binding to bacterial cells. Only background fluorescence was observed after incubation with GFP alone. Therefore signals detected in samples after incubation with LssSH3b_GFP could be attributed to specific binding of LssSH3b but not of GFP to the bacterial cell walls (Fig. 6). This was also confirmed by microscopic observations which demonstrated that the LssSH3b domain binds specifically to the cell walls of *S. aureus*. No fluorescence was observed either on the surface of *E. coli* after incubation with the fusion protein or *S. aureus* incubated with GFP alone (Fig. S4).

The results confirm that LssSH3b domain binds specifically to *S. aureus* and that the efficiency of the binding depends on the length and composition of the cross-bridges, as illustrated by decreased level of fluorescence detected in assays with *S. aureus* strain *femB* and *femAB* with shortened cross-bridges (3 or 1 glycine, respectively) and strain TF5311, in which one of the glycines in the cross-bridge is replaced by serine (Fig. 6).

We have engineered variants of LssSH3b domain with predicted wider pentaglycine binding groove by substituting asparagine 405 (Asn405) and tyrosine 472 (Tyr472) by alanines. The introduced substitutions reduced binding by approximately 20–30% as compared to the wild type domain (Fig. 6). The impaired binding of the WT LssSH3b to *femB* mutant of *S. aureus* was not rescued by the single substitution introduced while double one rescued the negative effect of single substitutions and bound *S. aureus femB* mutant with the same efficiency as the WT protein. A similar effect was observed for strain with serine introduced in the cross- bridges. The only case of improved binding was observed for a double LssSH3b substitution and an *S. aureus* strain with single glycine in the cross-bridges (*femAB)* as compared to the WT *S. aureus* (8325-4 strain).

### Effect of amino acid substitutions on lysostaphin activity

To test the relevance of the selected residues in LssSH3b domain for the activity of lysostaphin the corresponding single and double substitutions, N405A and Y472A, were introduced in the background of mature enzyme.

**Figure 7:**
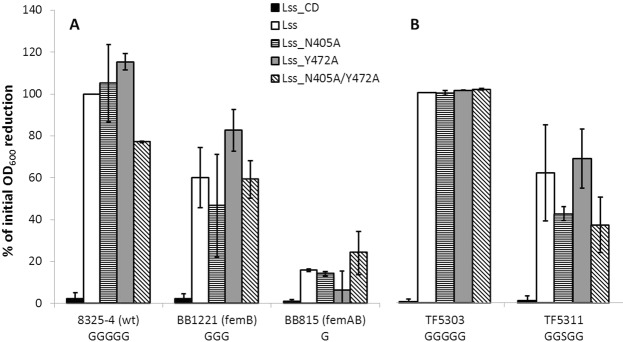
Lytic activity of lysostaphin with substituted residues in SH3b domain. (**A**) 10 nM enzymes activity was measured within 2 h at room temperature, (**B**) 100 nM enzymes activity was measured within 1 h at room temperature. Results present the percentage of reduction of initial OD_600_ (mean value from 3 independent experiments in triplicates, error bars represent standard deviation).

When the standard concentration of purified enzymes (100 nM) was used, no differences were observed in lysis of the *S. aureus* with different length of cross-bridges between the native and lysostaphin with introduced substitutions. In the next step the concentration of the enzymes was reduced 10 fold to detect subtler differences. Even in such stringent test conditions variants with single substitutions behaved the same as the WT protein. Y472A variant turned out to be slightly more efficient in lysing *femB* mutant strain than the WT enzyme and N405A/Y472A variant was more active against *femAB* mutant strain (with single glycine in the cross-bridges). We concluded that although the residues Asn405 and Tyr472 are involved in peptidoglycan binding, their replacements with alanine do not affect lytic activity of the mature lysostaphin significantly, at least in the assays and conditions used (Fig. 7A).

Apart from revealing the role of selected residues on peptidoglycan binding we were also expecting that these changes might expand the activity of lysostaphin resistant strains having serine in the cross-bridges. No activity of the WT or any of the variants was detected when 10 nM enzymes were used in the assays with *S. aureus* strain TF5311 carrying *epr* gene responsible for introduction of serine(s) in the cross- bridges. Therefore the lytic tests were run with 100 nM lysostaphin and its variants, which all demonstrated the same, full activity toward the wild type strain (TF5303). Both WT lysostaphin and Y472A variant displayed approximately 40% decrease of the lytic activity towards the TF5311 strain, while in the case of variant carrying N405A substitution this efficiency dropped further 20%. These results clearly show that the effect of shortening the side chain of residue 472 side chain is rather negligible while substitution of asparagine N405 by alanine (N405A) impairs significantly the lysostaphin activity. None of the introduced substitution expanded the activity of the enzyme to lysostaphin resistant strain (Fig. 7B).

## Discussion

### Structural basis of pentaglycine specificity

Analysis of the structure explains the preference of the lysostaphin SH3b domain for glycine residues. Such preference could be reinforced indirectly, by requiring the bound peptide to be in a conformation only accessible to glycine residues. Alternatively, the preference for glycine residues could be enforced more directly, by steric clashes of the Cβ atom present in all other amino acids with residues of the SH3 domain. The question whether an additional Cβ atom could be accommodated in the crystal structures can be addressed for all five glycine residues in the ligand, whereas Ramachandran angles are only defined for Gly2, Gly3 and Gly4 for the pentapeptide. Gly1 would have defined Ramachandran angles in the context of a larger pentapeptide fragment. For Gly5, which is isopeptide-linked to the lysine side chain amino group of a stem peptide, at best an analogous measure to the Ramachandran angles could be defined.

For the first residue of the pentapeptide, *in silico* analysis places a potential Cβ atom too close to Tyr411. The shortest distance (2.3Å or 2.6Å, depending on which molecule of the asymmetric unit is analyzed) is clearly shorter than the sum of van der Waals radii of the CH2 and CH3 groups (1.7Å + 2.0Å = 3.7Å). The steric conflicts are thus sufficiently severe to be potentially biologically relevant according to a recent analysis of such clashes that allows for protein and ligand adaptive fit.

However, we note that the side chain of Tyr411 is not obviously anchored and may give way to some extent to make space for a larger residue.

In the crystal structure Gly2 adopts a rare left-handed α-helix conformation that is in principle also accessible to amino acids other than glycine. Modeling of a substitution of Gly2 to an alanine in this position introduces a Cβ atom close to Tyr411 (shortest distance ca. 2.8–2.9Å, depending on the molecule that is analyzed in the asymmetric unit).

Gly3 is placed in a region of the Ramachandran plot (ψ ~ 0, χ ~ 80°) that is favorable for glycine residues, but not accessible to other amino acids. Moreover, a Cβ atom on this residue would also come close to several atoms of Tyr472 (shortest distances 2.8–2.9Å). This is consistent with the observation that a change from glycine to serine in the peptidoglycan of lysostaphin-resistant staphylococci protects against lysostaphin activity and diminishes binding to the SH3b domain^14,23^.

Gly4 adopts a main chain conformation that is on the edge of the β-sheet region of the Ramachandran plot for all amino acids, but in the favored region for glycine. Any side chain on this residue would point towards solvent, and could therefore be easily accommodated.

The Gly5 main chain conformation is at the edge of the β-sheet region. A potential Cβ atom would be placed 2.7–2.8Å away from Tyr407. However, the Gly5 conformation is in part dictated by a salt bridge between the glycine carboxylate and the guanidino group of an arginine (Arg476) of a crystallographic neighbor. Moreover, the free carboxylate group would be replaced by a carboxamide in a larger peptidoglycan fragment. Therefore, the conformation of Gly5 may not be representative for the conformation of this residue in peptidoglycan.

In summary, we conclude that pentaglycine specificity appears to result from a combination of direct and indirect effects. The replacement of glycines in the pentapeptide by other residues would lead to clashes with N405 and Tyr472, but possibly also with Tyr407 and Tyr411. Tyr411 is conserved among the staphylolytic enzymes (lysostaphin, Ale-1, *S. aureus* autolysin, *S. aureus* phage Twort amidase, and *S. aureus* phage PVL amidase). Tyr407 is present in all members of the group with the exception of the PVL amidase. Indirect selection for glycine by selection of a main chain conformation only accessible to glycine is clear-cut in case of Gly3, but may also operate to a lesser extent for other glycine residues.

### Interactions beyond the pentaglycine

Both MST analysis and MD calculations shows that interactions between the short fragments of peptidoglycans (pentaglycine, stem peptide) are weak. Similarly, NMR titration experiments also indicated a milimolar value of K_d_ for pentaglycine binding to the lysostaphin cell wall binding domain^27^ and other bacterial SH3b domains^28,29^. In contrast, the affinities calculated for LssSH3b binding to PG fragment comprising two stem peptides bridged by pentaglycine (MD) and entire bacterial cells (Scatchart analysis) indicated much stronger interactions. Micromolar K_d_ values indicate strong interactions of the domain with the peptidoglycan network and intact bacterial cell walls and are in the range of affinities reported for other cell wall binding domains, like LysM^6,30^,^31^. The highly specific binding domains e.g. CBD from *Listeria* have K_d_ values calculated in pico- to nanomolar range^10^.

According to available crystal structures, the peptide binding grooves of bacterial and eukaryotic SH3 domains are in different locations. The eukaryotic binding groove is located on the opposite side of the domain, as compared to pentaglycine binding groove bacterial domain (Fig. S7). Moreover. it is covered by the extended N-terminal characteristic for bacterial SH3b domains and therefore not accessible for ligands. Therefore, we have attempted modelling of peptidoglycan binding beyond already characterized interactions with pentaglycine based on available structural information and binding pocket predictions.

The predicted alternative conformations of the stem peptides differ mainly in the binding mode of the cross-linked stem peptide. The structural strains within the PG-fragment caused by conformational changes of the stem peptides and further propagated towards the flexible bridge get defused by structural readjustments of pentaglycines. The dynamical evolution from one pose to another happened mostly *via* rotation around the C1-Cα bond of the Gly1 residue of the pentaglycine bridge from 0 to 180 degrees and formation of *cis* peptide bonds which remains stable during a simulation process, even in high temperatures. The outstanding flexibility of the pentaglycine contributes to the PG-fragment’s extraordinary ability to adopt *cis* conformations of the peptide bonds. *Cis* bonds constitute a very small fraction of all peptide bonds. Recently, there have been several reports on presence and relevance of *cis* configuration of isopeptide bonds in Gram-positive bacteria that may use such intramolecular crosslinks as a mode of stabilization of cell surface structures and proteins^32–37^.

Interestingly, these strong changes in the geometry of the ligand did not correspond to any drastic structural changes in the SH3b domain. The only perceivable changes concerned the rearrangement of the non-bonding interactions within the SH3b-PG fragment complex, mostly the hydrogen bonds.

In summary, our modelling defines possible stable poses of the PG fragment bound to the SH3b domain, which map the area of interactions between the domain and the peptidoglycan stem peptides and reveal possible mechanisms of structural adaptation of the ligand by bond rotations and *cis* bond formation. Our predictions are similar to the ones published recently, although in both cases the predicted regions of stem peptides interactions do not overlay with the regions postulated based on NMR studies^27^ (Fig. S8).

### Substrate selectivity versus binding affinity

The main concern related to application of lysostaphin as an antimicrobial is resistance, which already exists in nature. The mechanism of resistance relies on substitution of the glycine(s) from the cross-bridge by serine(s). The lysostaphin producer – *Staphylococcus simulans* has such cross-bridges as a natural defense mechanism against lysostaphin.

Substrate selectivity of lysostaphin SH3b domain was confirmed by our assays with synthetic peptides, which demonstrated clearly that SH3b domain binds to pentaglycine while binding to GGSGG was not detected with the method and conditions used. However, when the SH3b was tested on intact cells of *S. aureus* strain TF5311 carrying *epr* gene responsible for lysostaphin resistance binding was substantially inhibited, but not completely abolished. A similar extent of binding inhibition of peptidoglycans with serine modified cross-bridges was observed previously for both domains of lysostaphin and zoocin^38,39^, as well as in the case of Ale-1 binding domain^8^. These differences in binding to isolated pentapeptides and peptidoglycans/cells may be attributable to the heterogeneity of the cross-bridges in the strains used, as it cannot be excluded that there are still some pentaglycine bridges present in the cell walls which serve as receptors for the SH3b domain. In fact, *S. aureus* strains carrying the *epr* gene, such as TF5311 used in this studies, have been shown to have heterogeneous cross-bridges including fraction of pentaglycine^40^. Alternatively, loss of binding of SH3b domains to serine containing cross-bridges may be at least partially overcome by interactions of the domain with other parts of the peptidoglycans, which might not define specificity but could play a role in determination of binding affinity. Similarly to our observations, substitution of the asparagine in SH3b domain from LysGH15 lysine corresponding to N405 in lysostaphin SH3b domain had a significant effect on peptidoglycan binding which was much less pronounced in the lytic activity assays^28^.

### Mature lysostaphin – binding and activity

**Figure 8:**
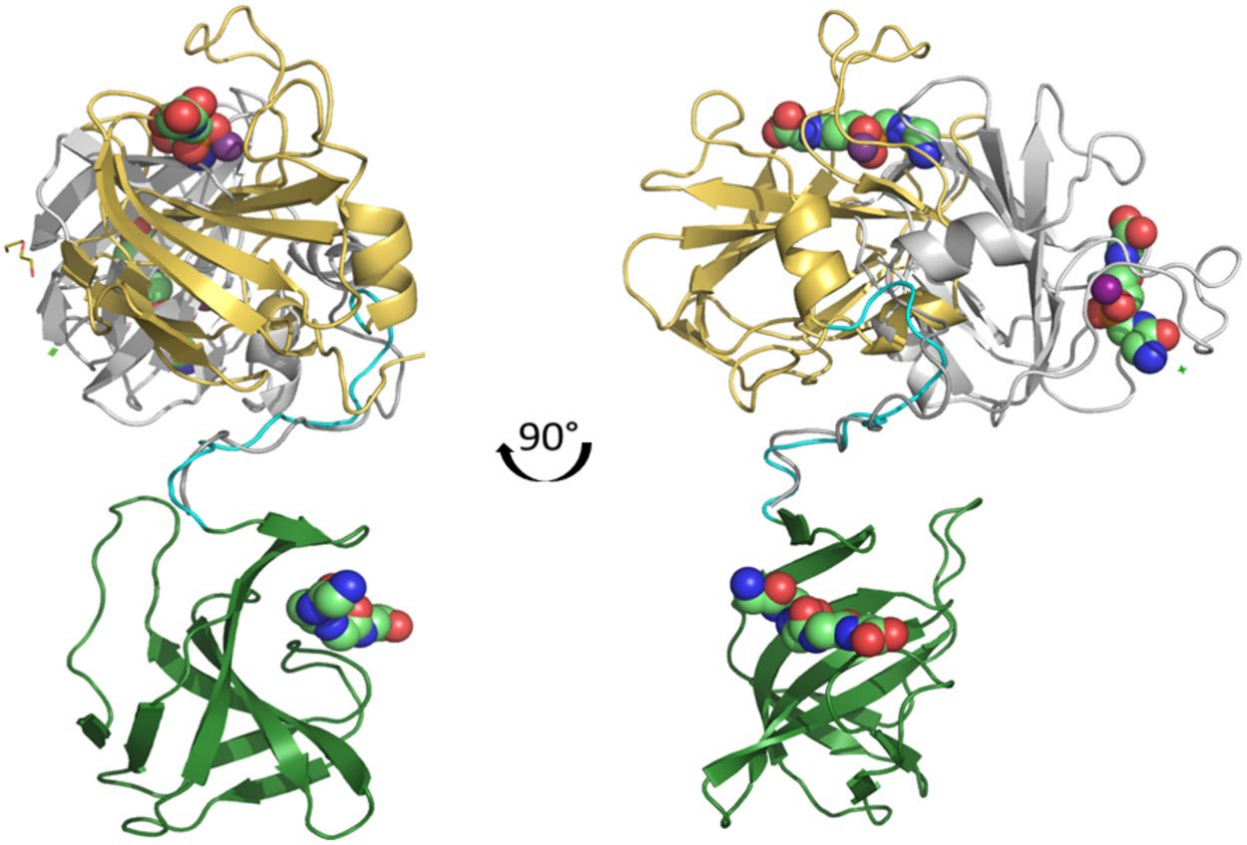
Binding of mature lysostaphin to pentaglycine. Two molecules in the asymmetric unit of the mature lysostahin (PDB:4LXC) were superimposed to show the flexibility of the domains. The catalytic domains (yellow and gray) differ in orientation by almost 100°. Catalytic zinc ion shown as a ball and SH3b domains are green. Moreover, the structure of mature lysostaphin was superimposed with ligands from complex structures of LytM catalytic domain with substrate analogue (PDB:4ZYB) and LssSH3b domain with pentaglycine (PDB:5LEO).

When superimposed on the crystal structure of lysostaphin the two pentaglycines from the complex of catalytic domain of LytM with substrate analogue^41^ and the SH3b complex bind in approximately antiparallel orientation and are located on opposite sides of the protein molecule (Fig. 8). The NMR structure of lysostaphin solved recently (PDB:5NMY) shows even more flexibility of the linker and the whole range of positions the two domains can adopt in relation to each other^27^.

It has been demonstrated that the isolated catalytic domain of lysostaphin is less effective in lysing staphylococcal cells compared to mature lysostaphin, where it is accompanied by SH3b domain and that the activity can be detected only in low ionic strength conditions^16,17^. This indicated that decreased efficiency in cell lysis is due to impaired binding of the separate domain. Though it has been suggested that pentaglycine is the element of the staphylococcal cell wall necessary for SH3b binding^13^, we have demonstrated that this domain interacts also with other elements of the staphylococcal peptidoglycans. The SH3b domain does not only determine lysostaphin specificity, it contributes also significantly to the binding affinity of the entire enzyme which results in increased lytic efficiency. The anchoring of the enzymes to peptidoglycans in a specific location might lead to punctual lysis pattern which should deteriorate bacterial cell walls more effectively than bond cleavage scattered all over peptidoglycans^18,42^,^43^. As can be concluded from the analysis of the mature lysostaphin structure, the presence of a very flexible linker allows the catalytic domain to reach several cross-bridges when anchored to PG by the SH3b domain^18,27^.

## Materials and Methods

### Protein overexpression and purification

A DNA fragment encoding residues 402–493 of lysostaphin was cloned into a pET15b expression vector using *Nco*I and *Xho*I restriction sites. The protein was overexpressed in *E. coli* BL21(DE3) in LB medium by induction with 0.1 mM IPTG for 6 h in 25 °C. The cell pellet was suspended in 20 mM Tris-HCl pH 7.0, 1 M NaCl, 10% glycerol and disrupted with a Constant Cell Disruption System (Constant Systems Ltd.). The cell lysate was dialyzed against 20 mM Tris-HCl, pH 7.0, 50 mM NaCl. SH3b was purified by ion exchange chromatography on WB40S resin (Bio-Works, Uppsala, Sweden), eluted with about 0.5 M NaCl in 20 mM Tris buffer pH 7.0, followed by gel filtration on Superdex 75 column (GE Healthcare) in 20 mM Tris-HCl, pH 7.0, 50 mM NaCl buffer.

### Protein crystallization and structure determination

Purified Lss SH3b (70 mg/ml) was preincubated with 17 mM pentaglycine for 30 min at room temperature prior crystallization. Crystals were formed in crystallization buffer from Morpheus Screen (Molecular Dimensions) composed of Buffer system 3 (0.1 M Tris (base), Bicine buffer, pH 8.5), 0.09 M NPS (NaNO_3_, Na_2_HPO_4_, (NH_4_)SO_4_), 37.5% MPD (racemic), PEG 1000, PEG 3350 within two weeks. Crystallization trials were carried out by the vapour diffusion method in sitting drops at 18 °C. Crystals were cryoprotected directly from the crystallization drop.

The datasets were collected at beamline ID29 of European Synchrotron Radiation Facility (ESRF, Grenoble, France). The best crystals diffracted to around 1.6Å and the data were processed and scaled with XDS^44^. The statistics of the diffraction data are summarized in Table S1. The orthorhombic crystals that belong to space group P2_1_2_1_2_1_ contained two SH3b domain-pentaglycine complexes in the asymmetric unit. Structure was solved by molecular replacement with the fragment of previously published lysostaphin structure (4LXC) covering the cell wall binding domain (residues 402–493)^18^ using Phaser-MR module in Phenix^45^. The model was completed by the manual addition of the pentaglycine molecules. Interactive model building was performed in COOT^46^ and refinement with Phenix^45^. Structure validation was carried out using Molprobity analysis^47^. Structural analyses, including superpositions, and structural figures were prepared in Pymol (http://www.pymol.org). The r.m.s.d. for chains A and B (SH3b domain) is 0.61Å for all atoms, r.m.s.d. between pentaglycines (chain G and chain H) was 0.22Å for all atom). As the two complexes in the refined model have nearly identical conformation they can therefore be described together.

### Mutagenesis

Lysostaphin SH3b domain variants with single residue substitutions: N405A and Y472A were generated by PCR-based site-directed mutagenesis according to the manufacture protocol with Phusion high-fidelity DNA polymerase (Thermo Scientific) using pET15b_SH3b plasmid as a PCR template. Mutations were confirmed by sequencing. The proteins with substitutions introduced were overexpressed and purified using the same protocols as for WT protein.

### GFP fusion

In order to express GFP tagged protein, lysostaphin SH3b domain was cloned into XhoI/BamHI site of pWALDO vector^48^ and LssSH3b_GFP fusion protein with C-Histag sequence was overexpressed in *E. coli* BL21(DE3) under T7 promoter. The protein was expressed in LB upon induction with 0.1 mM IPTG at 30 °C for 18 hours. The collected cells were sonicated and the lysate was applied on Ni+ column (HiTRAP, GE Healthcare). The imidazole gradient was run from 10 mM to 0.5 M in 20 mM Tris-HCl pH 8.0, 1 M NaCl, 10% glycerol. Histidine tagged LssSH3b_GFP protein eluted at 0.2 M imidazole was further purified by gel filtration on Superdex 75 column (GE Healthcare) in 20 mM Tris-HCl pH 8.0, 0.2 M NaCl, 10% glycerol. The gene for control GFP protein was cloned into NdeI/XhoI site of pET22b vector and GFP protein with C-Histag sequence was overexpressed in *E. coli* BL21(DE3) under T7 promoter. The expression was induced in LB medium with 1 mM IPTG at 28 °C for 18 hours and GFP protein was purified using the protocol optimized for LssSH3b_GFP fusion protein described above.

### Fluorescent binding assay

Cells of *S. aureus* grown in TBS and *E. coli* grown in LB were harvested from overnight or from log phase cultures by centrifugation and resuspended to OD600 of 20 in PBS. 130 µl of the bacterial suspension was mixed with various amounts of LssSH3b_GFP ranging from 1 to 23 µg in a final volume of 150 µl. After 15 minutes incubation at room temperature the reaction mix was centrifuged. Bacterial cell pellets were washed twice with 100 µl of PBS and finally resuspended in 50 µl PBS. Fluorescence was measured in 50 µl of suspension, supernatant and resuspended palettes. As controls mixtures of staphylococcal cells incubated with GFP and *E. coli* cells mixed with LssSH3b_GFP or GFP were used. The fluorescence intensity was measured in Tecan Infinite M1000 plate reader in 96 well plates using excitation at 480 nm and emission at 508 nm. All experiments were repeated at least twice.

### Microscopy

Cells of *S. aureus* 8325-4 and *E coli* from overnight culture were harvested by centrifugation and resuspended to OD_600_ of 20 in PBS. 40 μl of the bacterial suspensions were mixed with 15 μg of LssSH3b_GFP fusion protein or 10 μg of GFP. After 15 minutes incubation at room temperature cells were sedimented by centrifugation, washed twice with PBS and finally resuspended in 50 μl of PBS. Bacterial cells were observed using Eclipse Nikon 80i fluorescent microscope.

### Microscale thermophoresis

Microscale thermophoresis experiments were carried out using Monolith NT.115 (NanoTemper Technologies GmbH, Germany). LssSH3b was labeled with the NT-647-NHS fluorescent dye according to the supplied labeling protocol Monolith NT™ Protein Labeling Kit. A series of dilutions of pentaglycine were prepared using buffer solution containing 100 mM glycine pH 10 and 100 mM NaCl. The enzyme was prepared in the same buffer. The solution of labeled LssSH3b was mixed 1:1 with different concentrations of pentaglycine yielding a final concentration of 10 nM of the enzyme and pentaglycine in the range of final concentrations between 0.2 and 17.0 mM. After 10 min of incubation, the NT.115 capillaries (NanoTemper Technologies) were filled with the enzyme/peptide solution and the thermophoresis was measured at a LED power of 100% and an MST power of 60%. The K_d_ was determined by nonlinear fitting of the thermophoresis responses using the NTAnalysis software.

### Scatchard analysis

S. aureus cells in PBS buffer were mixed with LssSH3b_GFP (2 to 5 µM) and incubated for 5 min at room temperature. The number of cells was determined before for each experiment and was in the range from 3,31*10^8^ to 1,2*10^9^ cells/ml. The mixture was centrifuged and free LssSH3b_GFP was obtained in the supernatant, while bound fraction was collected in the cell palette. The fluorescence of both fractions was measured using Tecan Infinite M1000 plate reader in 96 well plates using excitation at 480 nm and emission at 508 nm. All experiments were done in triplicates and repeated three times. The ratio of bound to free LssSH3b_GFP was plotted as a function of the concentration of bound. The K_d_ was determined by Scatchard analysis. A plot of free versus bound LssSH3b_GFP yields a straight line of slope (−1⋅Kd–1) which intercept on the X-axis indicate the value of maximum concentration of bound LssSH3b_GFP.

### Turbidity reduction assay

Various strains of *S. aureus* were cultivated in tryptic soy broth (TSB) to an optical density of approximately OD_595_ ~ 0.6. Then the bacterial suspension was centrifuged for 10 minutes at 2500xg and the resulting bacterial pellet suspended in appropriate buffer. The optical density of the bacteria was adjusted to OD_595_ = 1.0. The test was performed on 96-well plates. If not otherwise, 100 nM enzyme were used in assays and the effect of their activity was observed in iMark™ Microplate Absorbance Reader (Biorad) at 595 nm. The measurements were made every 10 minutes for one hour at room temperature. Additional controls were run for the enzymes used in the assays: the MS confirmed that all enzyme preparations were homogenous and that the proteins used in the assays carried correct substitutions.

### Binding pockets prediction

Binding pockets of the lysostaphin SH3b protein domain (PDB ID: 5LEO) were prediction and their druggability was assessed using the DogSiteScorer webserver^25^ on the basis of the “chain B” representation of the 5LEO crystallographic structure.

### Molecular modeling of the SH3 domain-peptidoglycan complex

The SH3b domain’s structure was processed using Chimera v1.11.2^49^: all heteroatoms were removed and a dockprep protocol with its default settings was applied. The simplified (i.e., lacking the glycan backbone of alternating units of N-acetylglucosamine and N-acetylmuramic acid) peptidoglycan fragment (PG fragment, Figs S5, S6) was modeled on the basis of the crystallographically determined structure of the pentaglycine complexed with the SH3b protein domain (PDB ID: 5LEO). The three-dimensional model of structure of this PG fragment was modeled by merging the missing oligopeptides (*i.e*., bridge link and cross link in the Fig. 5, pane A) with the pentaglycine template followed by the optimizationwith the obminimize program of the Open Babel package v2.4.0^50^ using the MMFF94 force field. Set of 313355 conformers of the ligand with a frozen pentaglycine core were generated using confab application^51^, and scored with the rDock dock_solv scoring function^52^. The top scoring 50% poses were selected using KNIME Analytics Platform^53^, and clustered with the root-mean-square deviation (RMSD) cutoff of 6Å. The five most populated clusters were then inspected visually and two representative poses were selected to reflect the potential binding modes of the ligand (see: Results and discussion). They were further fine-tuned manually to maximize the hydrogen bond interactions with the SH3b protein domain and became the starting conformers of the ligand for the further modelling at Molecular Mechanics level. The SH3b complex with GGSGG peptide was determined using molecular docking with rDock docking program with dock_solv scoring function.

### Molecular dynamics study of the SH3b protein domain - PG fragment complex

DFT calculations at the HandHLYP/6–311 g(d,p) level^54,55^ have been performed to model the molecular properties and geometry (atomic charges, bond lengths, angles and torsions) of the ligand using ORCA v4.0.1 program package^56^. [D3] These results were used to build the ligand unit and adjust the AMBER99SB-ILDN force field^57^ for Molecular Mechanics (MM) calculations of the protein - ligand complex, performed using the GROMACS v5.0.7 Molecular Dynamics (MD) package^58,59^. The force field structural parameters have been modified according to the DFT results^54^ to reproduce more realistically molecular properties of the studied system and to obtain more reliable morphologies of the protein – ligand complex from the MM simulations. To validate the correctness of the two previously selected poses from the initial molecular modelling, a series of MM calculations in explicit solvent (TIP4P) [D7] have been performed. The starting configuration included SH3b protein domain with: (i) the ligand in the two selected poses, (ii) pentaglycine positioned as in the 5LEO structure, and (iii) both oligopeptides positioned as in the pose with the ligand bound tightly with the SH3b domain. The procedure was to minimize the energy (at 0 K) of the starting configurations. Then, for each stable optimized structure, a set of dozens short (few ns) quenched Molecular Dynamics (NVT followed by NpT) simulations (at 600 K and 300 K) were performed. The simulations at 300 K have been employed to check the stability of the ligand-SH3b complex, while at 600 K used to explore the conformational space of the ligand upon interaction with the SH3b domain. The procedure was terminated once the most stable poses of ligand were identified.

## Supplementary information

Supplementary information
