# Supplementary material for: Structural bases of peptidoglycan recognition by lysostaphin SH3b domain
Source: Sci Rep. 2019 Apr 12;9:5965. doi: 10.1038/s41598-019-42435-z (PMC6461655; doi:10.1038/s41598-019-42435-z)
Supplement: Supplementary file 1 — Supplementary information [file 41598_2019_42435_MOESM1_ESM.pdf]

Supplementary materials to manuscript:

Structural bases of peptidoglycan recognition by lysostaphin SH3b domain.

Paweł Mitkowski[1]*, Elżbieta Jagielska[1]*, Elżbieta Nowak[1], Janusz M. Bujnicki[1,2], Filip Stefaniak[1], Dorota Niedziałek[3], Matthias Bochtler[1,3] and Izabela Sabala[1#]

[1] International Institute of Molecular and Cell Biology in Warsaw, Poland

[2] Laboratory of Bioinformatics, Institute of Molecular Biology and Biotechnology, Faculty of Biology, Adam Mickiewicz University, Poznan, Poland

[3] Institute of Biochemistry and Biophysics Polish Academy of Sciences, Warsaw, Poland

**equally contributing authors*

[#]corresponding author

e-mail: izabela@iimcb.gov.pl

Ks. Trojdena 4, 02-109 Warsaw, Poland

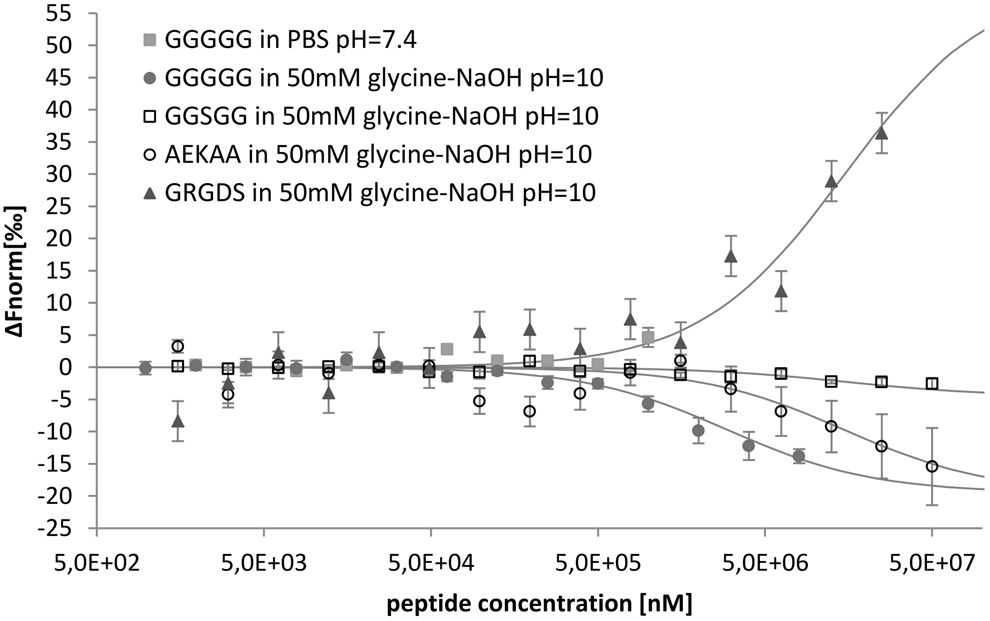

Figure S1. MST analysis of LssSH3b domain interactions with various peptidoglycan fragments.

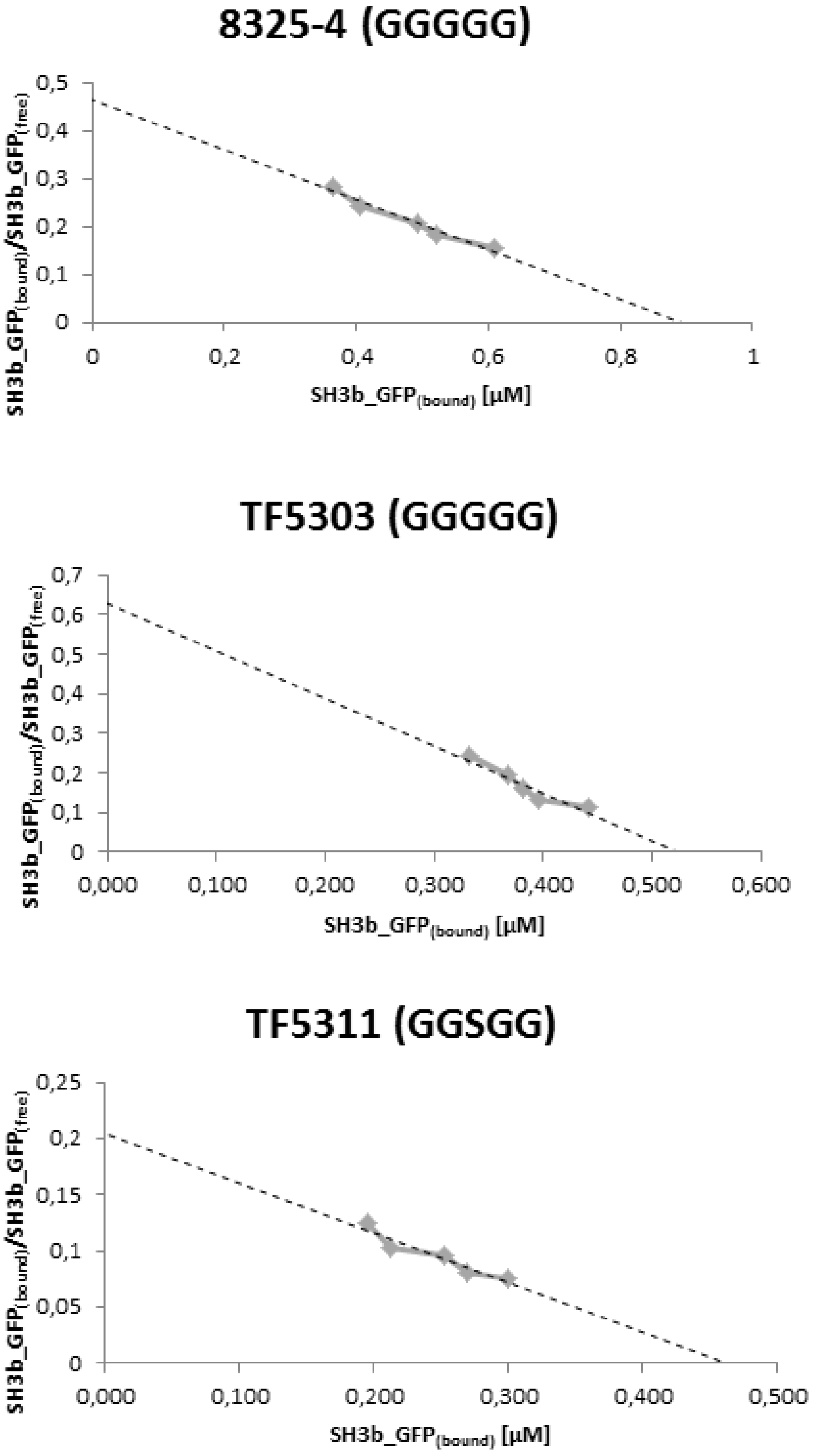

Figure S2. Examples of Scatchart analysis for binding of LssSH3b_GFP to various *S. aureus* strains. The ratios of concentrations of bound ligand to unbound ligand are presented versus the bound ligand concentration, from which the K_d was calculated.

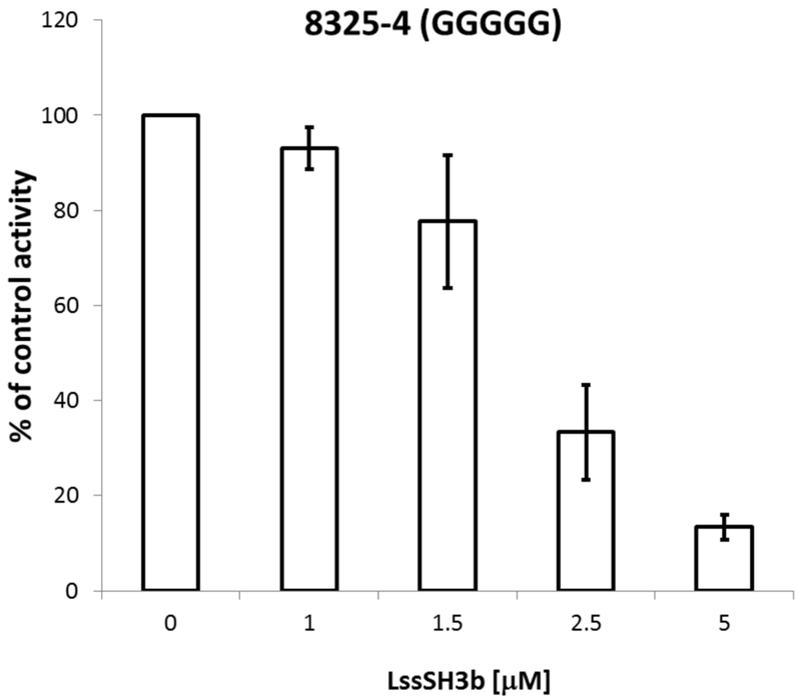

Figure S3. Lysostaphin activity inhibited by increasing concentration of SH3b domain preincubated with *S. aureus* 8325-4 strain. The activity was measured in turbidity reduction assay within 1h at room temperature. The plots represent % of control activity of 100 nM mature lysostaphin without LssSH3b domain preincubation.

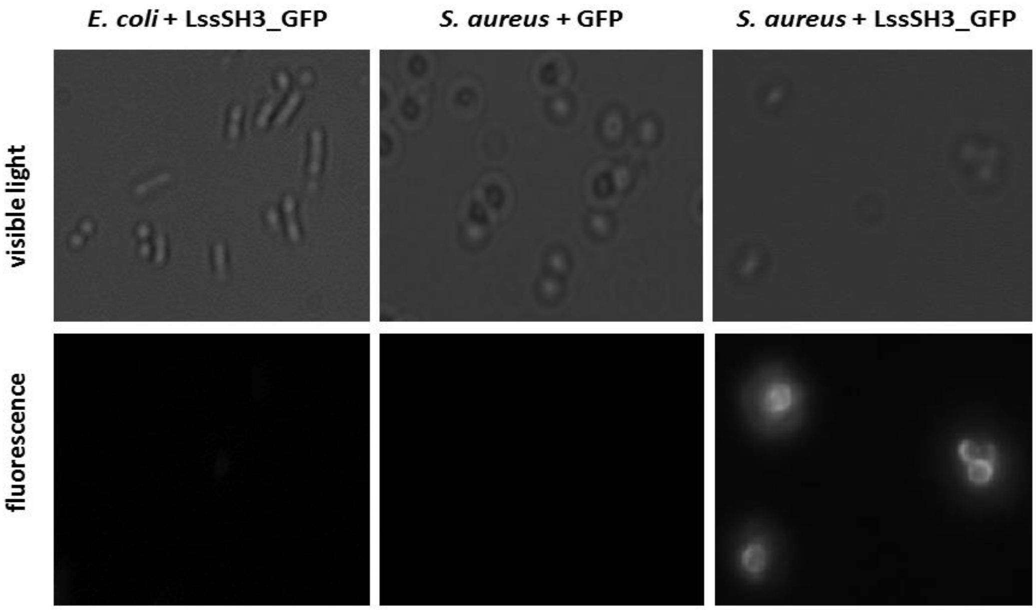

Figure S4. Fluorescence microscopy illustrating specific binding of lysostaphin SH3b domain fused to GFP to *S. aureus*.

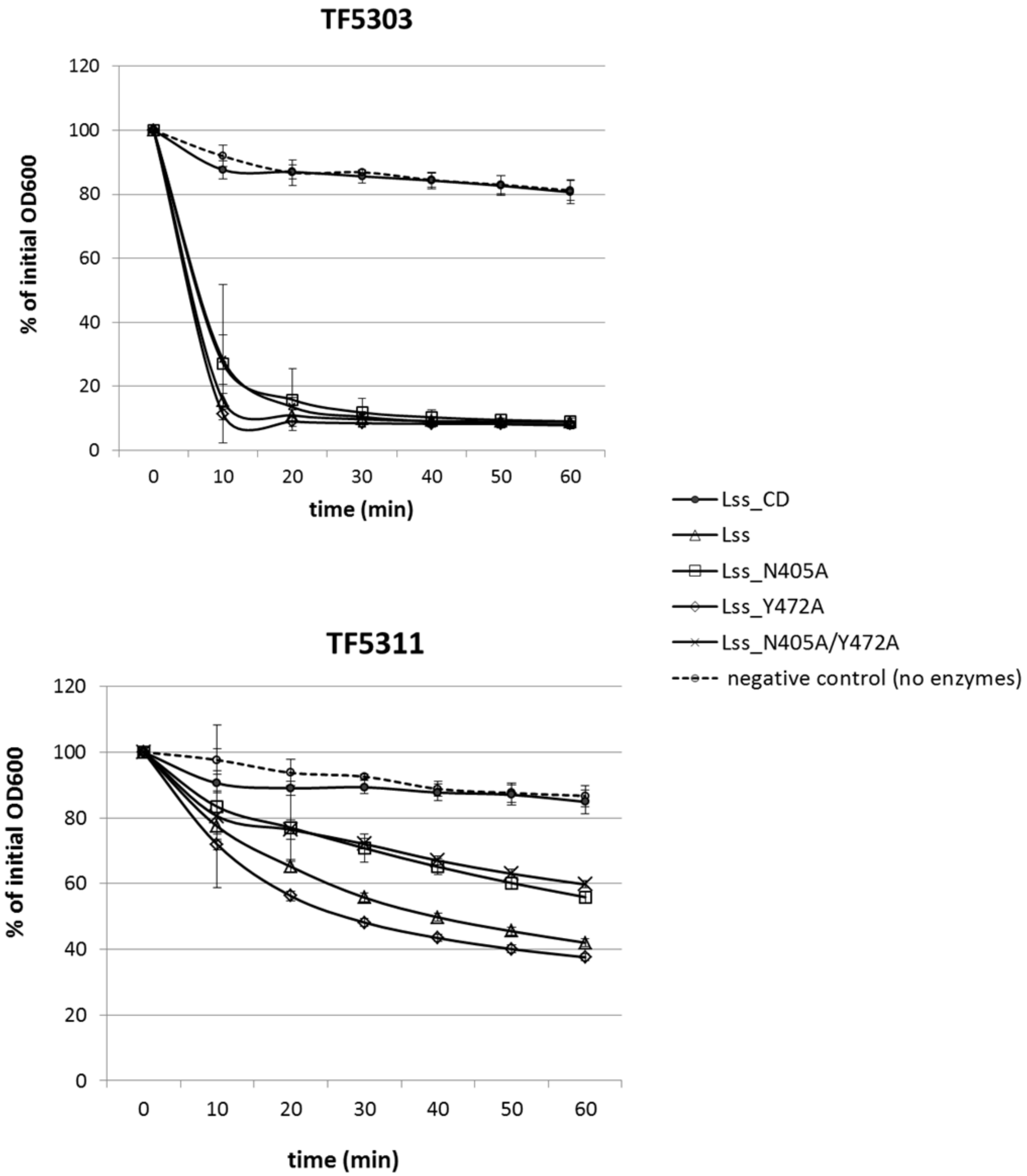

Figure S5. Lysostaphin activity and its SH3b domain variants (100 nM enzymes) measured in turbidity reduction assay within 1h at room temperature. The plots present % of OD_{600} reduction with time while the enzymes were applied on TF5303 or TF5311 *S. aureus* strains.

[H][C@@](CCC(=O)N[C@@]([H])(CCCCN)C(=O)N[C@H]©C(=O)NCC(=O)NCC(=O)NCC(=O)NCC(=O)NCC(=O
)NCCCC[C@]([H])(NC(=O)CC[C@]([H])(NC(=O)[C@H]©N)C(N)=O)C(=O)N[C@H]©C([O-
])=O)(NC(=O)[C@H]©N)C(N)=O

Figure S6. The SMILES code of peptidoglycan (PGN) fragment structure used for modeling.

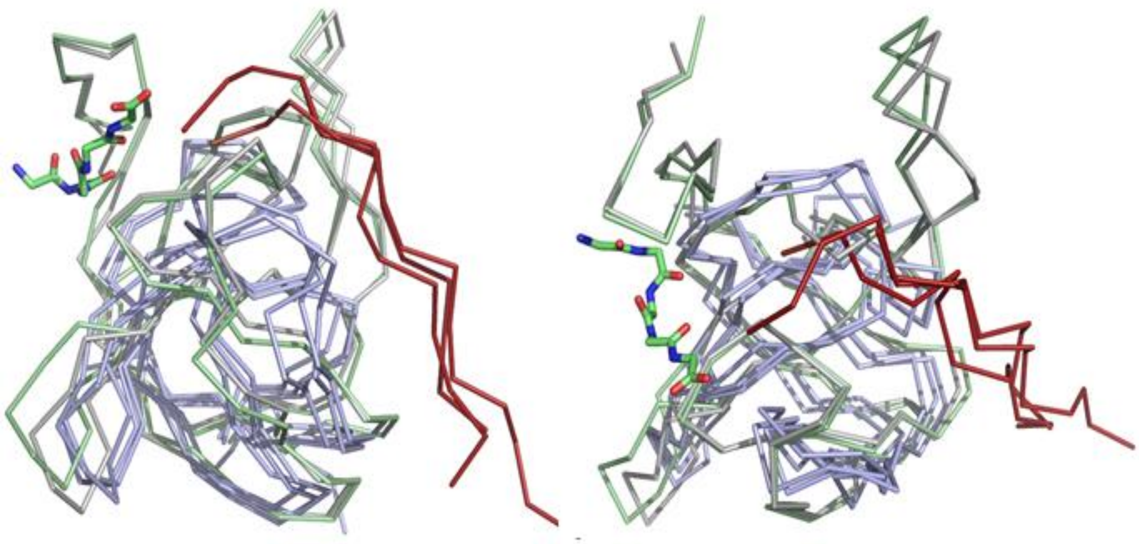

Figure S7. Superimposition of SH3 structures: eukaryotic (blue backbone with brick red ligands; PDB: 1CKA, 2OJ2, 5L23), Ale-1 (gray; PDB: 1R77) and lysostaphin in complex with pentaglycine (green backbone with stick representation of pentaglycine; PDB: 5LEO)

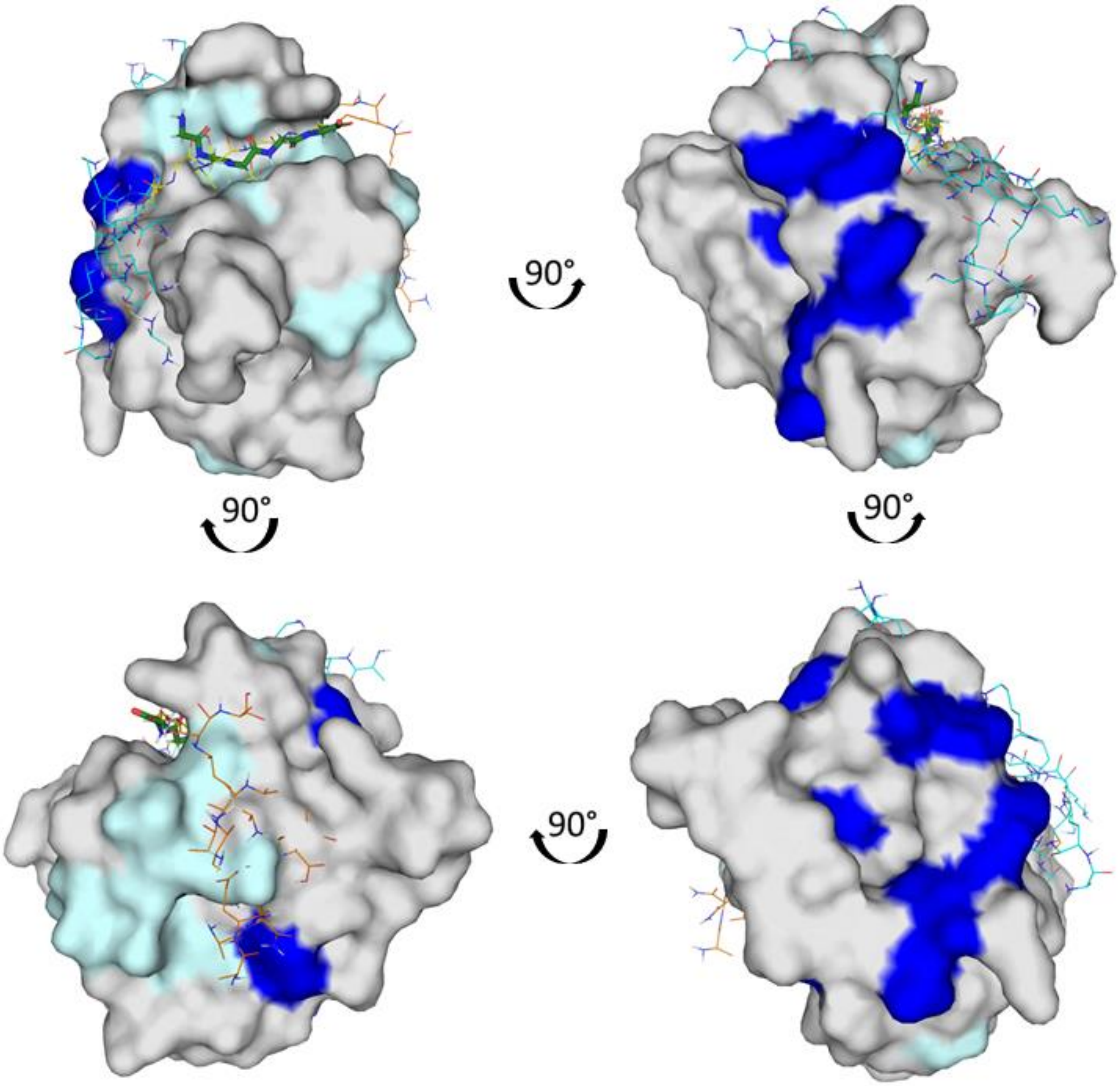

Figure S8. Mapping of experimental and modelled interactions between SH3b domain and peptidoglycan. K_d values calculated by Tossavainen et al. (Figure 5, A, B, C) [27]were mapped to the structure of SH3b domain (5LEO) with ligand, dark blue representing the higher affinity site and light blue the lower affinity site. Pentaglycine is shown in green, PG-fragment model obtained from MD studies is shown as in Figure 5.

Table S1. Statistics of data collection and refinement.

Data collection statistics	
Space group	$P2_12_12_1$
Cell dimensions	
a, *b*, *c* (Å)	50.4, 54.4, 63.2
α, β, γ (°)	90, 90, 90
Beamline	ID29 at ESRF
Wavelength (Å)	0.8726
Resolution (Å)	39.40-1.56 (1.56-1.66)
R_{meas} (%)*	24.3 (114.3)
$CC_{1/2}$*	99.3 (53.5)
$I/\sigma I$*	5.25 (1.28)
Completeness (%)*	98.9 (94.4)
Multiplicity*	4.7 (4.7)
V_M	2.08
Solvent content (%)	40.90%
Refinement statistics	
No. reflections	23461
$R_{work/}$ R_{free}**(%)	18.4/23.5
No. atoms	
Protein	1516
Ligand/ion	46
R.m.s deviations	
Bond lengths (Å)	0.007
Bond angles (º)	1.115
Ramachandran<br>allowed region (%)<br>favored region (%)	 2.11<br>97.89
Molprobity clashscore	2.91

Values in parenthesis are for the highest shell.
